# Liquid biopsy: circulating tumor DNA monitors neoadjuvant chemotherapy response and prognosis in stage II/III gastric cancer

**DOI:** 10.1002/1878-0261.13481

**Published:** 2023-07-04

**Authors:** Meng Zhang, Heli Yang, Tao Fu, Meizhu Meng, Yi Feng, Changda Qu, Zhongwu Li, Xiaofang Xing, Wenmei Li, Meiying Ye, Sisi Li, Zhaode Bu, Shuqin Jia

**Affiliations:** ^1^ Center for Molecular Diagnosis, Key Laboratory of Carcinogenesis and Translational Research (Ministry of Education/Beijing) Peking University Cancer Hospital & Institute Beijing China; ^2^ Gastrointestinal Cancer Center, Key Laboratory of Carcinogenesis and Translational Research (Ministry of Education/Beijing) Peking University Cancer Hospital & Institute Beijing China; ^3^ Department of Pathology, Key Laboratory of Carcinogenesis and Translational Research (Ministry of Education/Beijing) Peking University Cancer Hospital & Institute Beijing China; ^4^ Department of Gastrointestinal Translational Research, Key Laboratory of Carcinogenesis and Translational Research (Ministry of Education/Beijing) Peking University Cancer Hospital & Institute Beijing China

**Keywords:** circulating tumor DNA, gastric cancer, neoadjuvant chemotherapy, prognosis, response

## Abstract

A good response to neoadjuvant chemotherapy (NACT) is strongly associated with a higher curative resection rate and favorable outcomes for patients with gastric cancer (GC). We examined the utility of serial circulating tumor DNA (ctDNA) testing for monitoring NACT response and prognosis in stage II–III GC. Seventy‐nine patients were enrolled to receive two cycles of NACT following gastrectomy with D2‐lymphadenectomy. Plasma at baseline, post‐NACT, and after surgery, and tissue at pretreatment and surgery were collected. We used a 425‐gene panel to detect genomic alterations (GAs). Results show that the mean cell‐free DNA concentration of patients with clinical stage III was significantly higher than patients with stage II (15.43 ng·mL^−1^ vs 14.40 ng·mL^−1^). After receiving NACT and surgery, the overall detection rate of ctDNA gradually reduced (59.5%, 50.8%, and 47.4% for baseline, post‐NACT, and postsurgery). The maximum variant allele frequency (max‐VAF) and the number of GAs decreased from 0.50% to 0.08% and from 2.9 to 1.7 after NACT. For patients with a partial response after NACT, the max‐VAF and the number of GAs declined significantly, but they increased for patients with progressive disease. Patients with detectable ctDNA at baseline, after NACT, or after surgery have a worse overall survival (OS) than patients with undetectable ctDNA. The estimated 3‐year OS was 73% for the post‐NACT ctDNA‐negative patients and 34% for ctDNA‐positive. Patients with perpetual negative ctDNA before and after NACT have the best prognosis. In conclusion, ctDNA was proposed as a potential biomarker to predict prognosis and monitor the NACT response for stage II–III GC patients.

## Graphical abstract

Using a cancer‐specific panel and gene alterations analyses, we examined the utility of circulating tumor DNA (ctDNA) testing for monitoring neoadjuvant chemotherapy (NACT) response and prognosis in gastric cancer patients. The decline of the maximum variant allele frequency and the variant number suggested a good response. The persistence of ctDNA after NACT or surgery correlated with shorter overall survival.

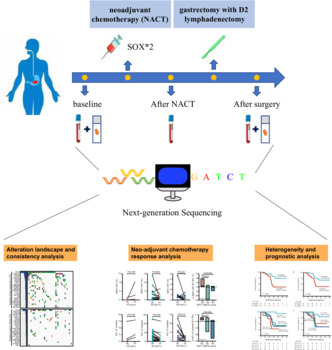

**Abbreviations**
ANOVA: analysis of variance
ATG‐Seq: Automated Triple Groom Sequencing
CAR‐T: chimeric antigen receptor T cell
cfDNA: cell‐free DNA
CNVs: copy‐number variations
CT: computed tomography
ctDNA: circulating tumor DNA
DFS: disease‐free survival
GAs: genomic alterations
GC: gastric cancer
HDR: homology‐directed repair
HR: hazard ratio
IGV: Integrative Genomics Viewer
indels: insertions and deletions
MAPK: mitogen‐activated protein kinase
MRD: molecular residual disease
MSI‐H: microsatellite instability
NACT: neoadjuvant chemotherapy
OS: overall survival
PD: progressive disease
PD‐1: programmed cell death 1
RECIST: Response Evaluation Criteria in Solid Tumors
S‐1: oral tegafur–gimeracil–oteracil potassium capsule
SD: stable disease
SNVs: single nucleotide variants
SOX: S‐1 plus oxaliplatin
SSA: single‐strand annealing
VAF: variant allele frequency

## 1 Introduction

Gastric cancer (GC) is the third leading cause of cancer‐related mortality both in China and worldwide [1, 2]. Despite its decline in incidence and mortality over the past five decades, GC is still prevalent in East Asia [3, 4]. Surgery is the first choice to treat GC [5]. But many patients lose the chance of surgery since it is often diagnosed at an advanced stage [6]. Neoadjuvant chemotherapy (NACT) is administered as an approach to ‘downstaging and downsizing’ a locally advanced disease before attempting curative resection [7]. Multiple studies have shown the ability of NACT to increase the curative resection rate and improve prognosis in patients with stage II–III GC [8, 9, 10]. Unlike in Western countries, doublet chemotherapy with the oral tegafur–gimeracil–oteracil potassium capsule (S‐1) is the mainstream NACT in Asia because of its high efficacy, safety, and convenient administration [11, 12, 13].

A good response to NACT is strongly associated with favorable patient outcomes. However, the response to NACT varies between individual patients, and only 20% to 37% of these patients achieve a pathologic response [14, 15]. Therefore, minimally invasive methods for early detection for monitoring ongoing therapy are urgently needed to improve risk stratification in patients with GC.

Circulating tumor DNA (ctDNA) containing tumor‐specific DNA variants can be found in the cell‐free component of peripheral blood in a proportion of patients with solid tumors [16]. The detection of ctDNA could identify targetable alterations, molecular residual disease (MRD), and emerging therapy resistance across multiple tumor types [17, 18]. However, the dynamic changes of ctDNA in the neoadjuvant setting in GC have not been reported. In this study, we evaluated the potential role of ctDNA as a biomarker in monitoring response to NACT and assessed the additive value of ctDNA to further stratify patients with residual disease to predict early metastatic recurrence.

## 2 Materials and methods

This study has been approved by the Beijing Cancer Hospital Medical Ethics Committee (2017YJZ38), and experiments were undertaken with the understanding and written consent of each subject. Seventy‐nine patients diagnosed with GC at Peking University Cancer Hospital from November 2017 to January 2020 were prospectively enrolled in the study (NCT number: NCT03425058 www.clinicaltrials.gov). The clinical preoperative stage (cTNM) was confirmed by endoscopic ultrasonography or computed tomography (CT) and pathology, respectively. All patients were scheduled to receive two cycles of S‐1 plus oxaliplatin (SOX) NACT indicated by the standard of care clinical guidelines and following total or subtotal gastrectomy with D2 lymph node dissection according to clinical evaluation. Peripheral blood was collected at prechemotherapy (P0), postchemotherapy (P1), and postoperation (P2). Core biopsies were obtained at the baseline (T0) and surgery (T1).

Response Evaluation Criteria in Solid Tumors (RECIST) version 1.1 criteria were used to assess response to NACT. Patient clinic features including sex, age, smoking and drinking history, differentiation degree, and Lauren subtype were recorded. All cases were followed up until October 2021. This study was approved by the Medical Ethics Committee of Peking University Cancer Hospital and performed according to the Declaration of Helsinki Principles. We obtained informed consent from all the patients in the study before enrollment.

### 2.1 DNA extraction and library construction

Plasma and matched lymphocytes were isolated within 2 h from 8 mL of peripheral blood stored in EDTA blood collection tubes by centrifuging at 1800 ***g*** for 10 min at 4 °C or room temperature. All samples were tested in a Clinical Laboratory Improvement Amendments (CLIA), International ISO 15189, College of American Pathologists (CAP), and European Molecular Genetics Quality Network (EMQN) certified genomic testing facility (Nanjing Geneseeq Technology Inc., Nanjing, Jiangsu, China). According to the manufacturer's instructions, DNA was extracted from plasma, lymphocyte, and tumor tissue using the QIAamp Circulating Nucleic Acid Kit (Qiagen), QIAamp DNA Blood Mini Kit (Qiagen), and QIAamp DNA FFPE Tissue Kit (Qiagen, Venlo, Hulsterweg, The Netherlands) respectively, and quantified by Picogreen fluorescence assays using the provided lambda DNA standards (Invitrogen, Waltham, MA, USA). DNA from lymphocytes and biopsies was sheared using a focused ultrasonicator (Covaris, Woburn, MA, USA). Library construction was performed in 96‐well plates (Eppendorf, Hamburg, Germany) with the KAPA Hyper DNA Library Prep Kit, containing end repairing, dA ligation, and dual‐index attaching. Genomic DNA libraries were amplified with HotStart Phusion Polymerase for 4–7 cycles before hybridization.

### 2.2 Target region capture and next‐generation sequencing

A commercial 425‐gene panel (Nanjing Geneseeq Technology Inc.) that covered all the CDS regions of the 425 genes and part of spanning introns was used for hybridization enrichment. The tested genes were shown in Table S1. The capture reaction was performed with Dynabeads M‐270 (Life Technologies, Carlsbad, CA, USA) and xGen Lockdown hybridization and wash kit (Integrated DNA Technologies, Redwood, CA, USA) according to manufacturers' protocols. Captured libraries were amplified by PCR for 6–8 cycles, purified using AMPure XP beads, and quantified by qPCR (KAPA). Libraries were normalized to 2.5 nm and pooled on the chip. Sequencing was carried out with the HiSeq 4000 (Illumina, San Diego, CA, USA) with 2 × 75‐bp paired‐end reads. The average depth was 300×, 1500×, and 6000× for normal blood controls, FFPE, and cell‐free DNA (cfDNA), respectively.

### 2.3 Sequencing data analysis and mutation calling

Sequencing reads in fastq format were generated by base calling using bcl2fastq (Illumina, Inc.). Quality control was applied with an open‐source software trimmomatic, by removing the terminal adaptor sequences and low‐quality data. Filtered reads were mapped to the reference human genome GRCh37 by BWA with default parameters. Duplicated reads were marked by Picard and de‐duplication reads were re‐aligned at intervals with mismatches and recalibrated base quality scores using gatk. varscan2 v2.3.9 was used to identify single nucleotide variants (SNVs), small insertions, and deletions (indels) with a minimum variant allele frequency (VAF) threshold set at 0.1%. SNVs/indels were annotated with ANNOVAR and manually checked with the Integrative Genomics Viewer (igv).

To sensitively and specifically detect low‐abundance mutations in cfDNA, a customized library preparation with a bi‐barcoding system called Automated Triple Groom Sequencing (ATG‐Seq) was applied to cfDNA samples [19]. To assemble a position‐ and base substitution‐specific background error database based on allele frequency and distinct supporting reads throughout the panel, we constructed a bioinformatics polishing pipeline by sequencing a pool of plasma samples collected from over 30 healthy donors. An alternation was considered as sequencing noise if its allele frequency and distinct supporting reads were not significantly higher than the corresponding background errors in the database. To minimize the errors from PCR, hybridization, damaging, sequencing, and contamination, and avoid mutations from nontumor sources in cfDNA, we conducted the following procedures: (a) we sequenced the cfDNA fragment at a depth of ~ 5000×, which produced redundant DNA molecules; (b) mapping positions and a bi‐barcode system were used to maximize the representative power of unique DNA molecules; (c) a duplex assisted decoder system was used to filter mapping and sequencing artifacts. Genomic DNA from the white blood cells of the buffy coat after plasma separation was also analyzed as the normal control sample for germline and clonal hematopoiesis mutation filtering. ctDNA positivity was defined as followed: (a) for the mutations detected in tissues, supporting reads ≥ 3 and the total reads ≥ 100. (b) for the mutations undetected in tissues, supporting reads ≥ 6 and the total reads ≥ 100.

Copy‐number variations (CNVs) were detected using a freely available software package adtex (http://adtex.sourceforge.net) with default parameters. Somatic CNVs were identified using paired normal/tumor samples for each exon with a cut‐off of 0.65 for copy‐number loss and 1.50 for copy‐number gain.

### 2.4 Statistical analysis

The relationship between the concentration of plasma cfDNA and clinical characteristics was examined by the Student *t*‐test or analysis of variance (ANOVA). The relationship between the detectable rate and clinical characteristics was examined by the chi‐square test or Fisher's exact test. To analyze the enrichment of genomic alterations (GAs) between different NACT efficacy groups, the frequencies of GAs were calculated at the gene level, and GA frequencies of more than 10% were reported. The following formula calculated the dynamic change in the max VAF or the number of GAs: Δmax‐VAF = max‐VAF (P1 − P0); Δnumber of GAs = number of GAs (P1 − P0). The Wilcoxon signed‐ranks and rank‐sum tests were used to compare the max‐VAF and the number of variants in ctDNA before and after NACT, and to compare the Δmax‐VAF and Δnumber of GAs among different cohorts of response to NACT, respectively. Survival was displayed using Kaplan–Meier curves generated by the SurvMiner r package and compared using the Gehan–Breslow–Wilcoxon test. Differences were considered statistically significant at a threshold of *P* < 0.05. All statistical analyses were performed using spss version 22.0 (IBM, New York, NY, USA), graphpad prism version 5.0 (GraphPad Software, San Diego, CA, USA) and r 3.4.0 is an open‐source software.

## 3 Results

### 3.1 Demographic and clinical features

**Fig. 1:**
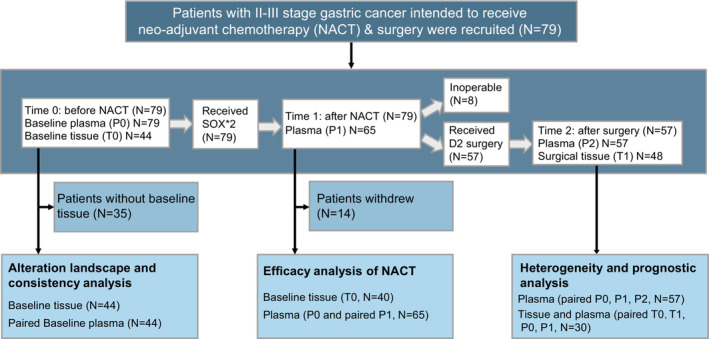
Overview of study design. Seventy‐nine patients diagnosed with II–III stage GC were enrolled in this study. All patients were scheduled to receive two cycles of oral tegafur–gimeracil–oteracil potassium capsule (S‐1) plus oxaliplatin (SOX) NACT following total or subtotal gastrectomy with D2 lymph node dissection according to clinical evaluation. Peripheral blood was collected at pre‐NACT (P0), post‐NACT (P1), and postoperation (P2). Tissue samples were obtained at the baseline (T0) and surgery (T1). Fourteen patients did not complete two cycles of NACT and eight patients withdrew for disease progress after NACT.

Patient enrollment and study design were presented in Fig. 1. The cohort included 56 males and 23 females and had a median age of 59 years (mean 60 years, IQR 52–65 years), which has been described in Table S2. Nearly a third of the patients had a smoking (35.4%) or drinking (25.3%) history, and they were all males. Of the enrolled patients, 72.2% were at stage III. Poorly differentiated adenocarcinoma (54.4%) and the intestinal type (44.3%) were more frequent than other pathological and Lauren types.

### 3.2 The concentration and detectable rate of baseline plasma cfDNA

As shown in Table S3, the mean cfDNA concentration of patients with clinical stage III was 15.43 ± 5.21 ng·mL^−1^, which was significantly higher than that of patients with stage II (14.40 ± 7.34 ng·mL^−1^). Meanwhile, patients in the progressive disease (PD) group had a significantly higher mean concentration of cfDNA than the stable disease (SD) group (20.68 ± 3.75 vs 13.57 ± 5.25, *P* = 0.033) but did not differ from the PR group (20.68 ± 3.75 vs 16.61 ± 6.54, *P* = 0.344). There was no difference in cfDNA concentration among other different clinical characteristics. The detectable rate was only different among the NACT efficacy groups. Plasma ctDNA was detectable in all PD patients, 44.4% of SD patients, and 72.0% of PR patients (Table S4). No significant association was observed between cfDNA concentration or detectable rate and other clinicopathological factors.

### 3.3 The genomic landscape and concordance in paired baseline tissue and plasma

**Fig. 2:**
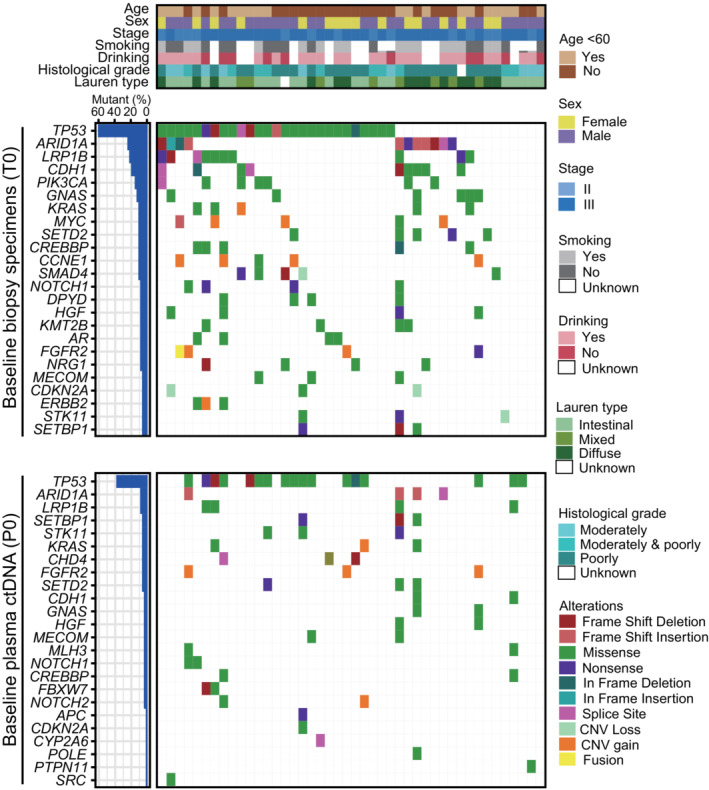
Summary of patients' characteristics and GAs landscape in paired tissue and plasma samples. Samples were classified by age, sex, clinical stage, smoking, drinking, histological grade, and Lauren types. GAs per sample in tissue was compared with those in paired plasma ctDNA.

Of these paired baseline plasma (P0) and tissue (T0) samples from 44 patients, 370 GAs were identified in 169 genes, with a median number of GAs of 8 for T0 and 5 for P0 samples. At least one somatic variant was identified in all T0 and 61.4% of P0 samples. One patient was identified with MSI‐High, who had a notably higher GA number than the other MSS patients (57 GAs for T0 and 21 GAs for P0). Of all the GAs, 33.8% were shared by T0 and P0, 56.2% were unique to T0, and 9.5% were unique to P0. *TP53*, *ARID1A*, and *LRP1B* were the most frequently altered genes in both T0 and P0 (Fig. 2). Amplifications were most enriched in *MYC*, *CCNE1*, and *FGFR2*. *HER2* amplification was seen in only 5% (2/44) of T0 samples but was undetectable in P0 samples. For baseline tissue, there was no significant difference in the genomic landscape between patients with detectable and undetectable ctDNA (Table S5).

We next compared the landscapes among patients with different responses to NACT (Fig. S1). For T0 samples, more frequent *ZNF217* amplification and *STK11*, *MECOM*, and *SETBP1* mutations were observed in the PD cohort. A higher incidence of mutations in *ROS1* and *CDH4* and amplification in *CCNE1* and *FGFR2* were observed in the PR cohort. In P0 samples, *SETBP1* and *STK11* were still the most frequent mutant genes in the PD cohort. No gene amplification was detected in ctDNA.

### 3.4 Dynamic changes of ctDNA and NACT treatment efficacy

The relationship between the dynamic change of concentration of cfDNA and the efficacy of NACT was first explored, and no significant difference was identified in cfDNA concentration before and after NACT (14.69 ± 6.07 ng·mL^−1^ vs 16.14 ± 9.84 ng·mL^−1^, *P* = 0.270). The overall ctDNA detectable rate of baseline and after NACT was 59.5% (47/79) and 50.8% (33/65), respectively. Among the 11 PR patients who had undetectable ctDNA after NACT, 5 (23.8%) were ctDNA negative at baseline, and 6 (28.6%) cleared ctDNA after NACT. Only one (5.0%) PR patient's ctDNA changed from negative to positive, and nine (42.9%) PR patients' ctDNA remained positive after receiving NACT. Of the 41 SD patients, 13 (31.7%) and 15 (36.6%) patients had consistently negative and positive ctDNA after NACT, respectively. Six (14.6%) SD patients' ctDNA changed from negative to positive, and seven (17.1%) SD patients' ctDNA changed from positive to negative. All 3 (100%) PD patients had continuously positive ctDNA before and after NACT (Table S6). However, there was no significant difference in ctDNA clearance between the different NACT efficacy groups (*P* = 0.231).

**Fig. 3:**
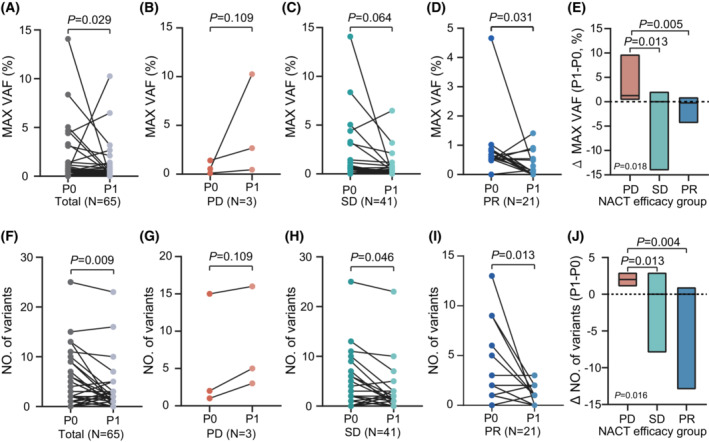
Dynamic changes of the maximum variant allele frequency (max‐VAF) and the number of variants of ctDNA before and after NACT. The Wilcoxon signed‐ranks and rank‐sum tests were used to compare the max‐VAF and the number of variants in ctDNA before and after NACT, and to compare the Δmax‐VAF and Δnumber of GAs among different cohorts of response to NACT, respectively. (A) changes of max‐VAF in all patients, (B) changes of max‐VAF in patients with PD, (C) changes of max‐VAF in patients with SD, (D) changes of max‐VAF in patients with PR, (E) the Δmax‐VAF among NACT efficacy groups, (F) changes of the number of variants in all patients, (G) changes of the number of variants in patients with PD, (H) changes of the number of variants in patients with SD, (I) changes of the number of variants in patients with PR, (J) the Δnumber of GAs among NACT efficacy groups.

We next explored the role of the dynamic change of ctDNA max‐VAF and the number of GAs in predicting the response to NACT. Overall, the max‐VAF of 65 patients with paired P0 and P1 decreased from 0.50% to 0.08% after NACT (Fig. 3A). Among them, the max‐VAF of patients in the PD group tended to increase (Fig. 3B). However, due to the small sample size, the results were not statistically significant. For patients with SD, most patients had a stable max‐VAF after NACT (Fig. 3C). The decline in the max‐VAF of PR group patients was notable (Fig. 3D). The Δmax‐VAF of patients with PD was remarkably higher than those of SD and PR patients (Fig. 3E). The number of GAs detected in ctDNA showed the same trend as that of max‐VAF (Fig. 3F). After receiving NACT, the overall number of GAs was less than it was in bassline (2.9 vs 1.7). The number of GAs of patients in the PD group tended to increase, but it was not statistically significant (Fig. 3G). For PR and SD patients, the number of GAs was significantly reduced after receiving NACT (*P* = 0.013 and *P* = 0.046) (Fig. 3H,I). The Δnumber of GAs in the PD group was remarkably higher than in the SD (*P* = 0.013) and PR groups (*P* = 0.004) (Fig. 3J).

### 3.5 Spatial and temporal molecular heterogeneity in GC

Genomic profiling of paired tissue and plasma samples from 30 patients before and after NACT was compared. Of the 30 patients, 80.0% (24) developed new GAs that were not present in the baseline tissue or plasma. As shown in Fig. S2a,b, significant inconsistencies in GAs were observed among baseline tissues, surgical tissues, and plasma samples before and after NACT. Only 7.7% of mutated genes and 4.4% of GAs were consistent in tissue and plasma before and after NACT. The results of GO analysis demonstrated that these GAs that vanished after NACT were particularly gathered in CD209, FLT3, down‐regulation of ERBB2 and ERBB3 signaling, regulation of TP53 activity through acetylation, acute myeloid leukemia, transcriptional regulation by AP‐2, and homology‐directed repair (HDR) through single‐strand annealing (SSA). GAs that appeared after NACT were mainly involved in constitutive signaling by AKT1 E17K in cancer, neoplasm of the genitourinary tract, and VEGFR2‐mediated vascular permeability (Fig. S2c).

### 3.6 ctDNA status and overall survival

**Fig. 4:**
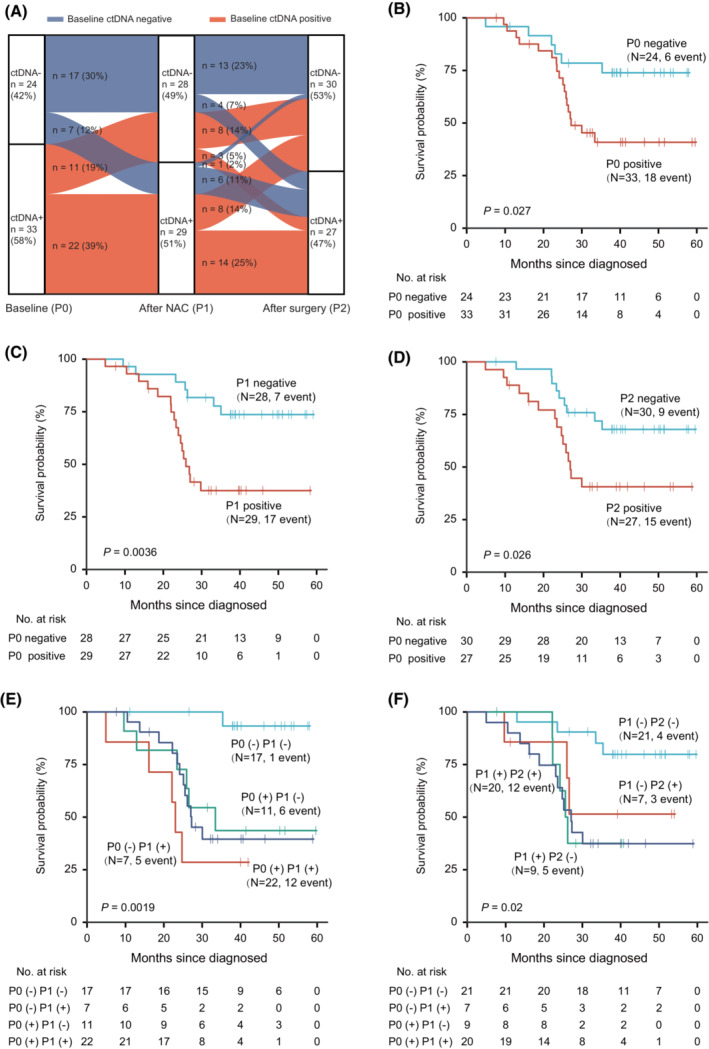
The dynamic changes of ctDNA status and Kaplan–Meier estimates OS according to ctDNA status. The Gehan–Breslow–Wilcoxon test was used for the comparison of survival curve. (A) The dynamic changes of ctDNA status after NACT and after surgery. OS analysis according to the ctDNA status at (B) baseline (P0), (C) 6 weeks after NACT (P1), (D) 4 weeks following surgery (P2), (E) combined ctDNA status at baseline and after NACT (P0 & P1), and (F) combined ctDNA status after NACT and surgery (P1 & P2).

As of 12 Dec 2022, 57 (72.2%) patients had completed two cycles of NACT and gastrectomy with D2‐lymphadenectomy. The median follow‐up was 32.4 months (range, 4.9–59.8 months). The dynamic changes in ctDNA status after NACT and surgery have been shown in Fig. 4A. The prognosis of patients with undetectable ctDNA was better whether at baseline, after NACT, or after surgery (Fig. 4B–D). The Kaplan–Meier estimates 3‐year overall survival (OS) was 74% and 38% for nonshedders and shedders at baseline, 73% and 34% for the after NACT ctDNA‐negative patients and ctDNA‐positive patients, and 68% and 38% for the after surgery ctDNA negative and ctDNA positive. When the ctDNA status at different time points was combined by pairs (P0 & P1, and P1 & P2), patients with a persistent undetectable ctDNA after NACT or surgery had a longer OS than patients with a detectable ctDNA (Fig. 4E,F). Among the four groups, patients with negative‐to‐positive ctDNA after NACT had the worst OS. There was no notable difference between the OS of patients with positive‐to‐negative and persistent positive ctDNA after NACT (Fig. 4E). Patients with positive‐to‐negative ctDNA after surgery had a survival advantage (< 22 months) over patients with persistent positive ctDNA in the short‐term. But in a long‐term perspective (>22 months), this advantage disappeared (Fig. 4F).

We next explored the relationship between the genotype of tissue or ctDNA and the OS time. For the low frequency of the most mutated genes, only mutated genes that were top five mutated genes either on tissue or on ctDNA, and a mutation number of more than five was analyzed. As shown in Fig. S3, patients with *KRAS* mutation in tissue had a worse OS. GAs of *TP53*, *LRP1B*, *ARID1A*, and *CDH1* in tissue had no significant effect on the OS. The presence of *LRP1B* and *CDH1* alterations in ctDNA had an adverse effect on the patient's OS. *TP53* and *ARID1A* alteration carriers showed the same trend (Fig. S4). It seems that the presence of GAs in ctDNA or not was more important than what genes were mutated in ctDNA.

### 3.7 Patient with microsatellite instability could not benefit from NACT

**Fig. 5:**
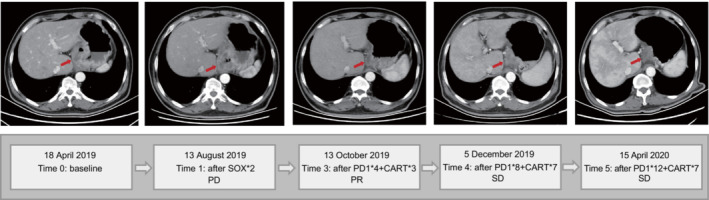
The abdominal computerized tomography (CT) image, therapeutic regime, and response to treatment of a patient with microsatellite instability. Contrast‐enhanced CT demonstrated that the gastric wall was thickened (red arrow) at baseline. After two cycles of NACT, the gastric wall was thicker than before, which indicated disease progression. And the patient could not receive surgical treatment for peritoneal metastasis. The treatment scheme was subsequently modified to immunotherapy. After four cycles of PD‐1 inhibitors and three cycles of CAR‐T administration, the gastric wall becomes thinner than after NACT, which indicated that the patients benefited from the immunotherapy. After 8 and 12 cycles of PD‐1 administration, the disease remained stable.

Patient #40, a 59‐year‐old male, was admitted to the Department of gastrointestinal surgery on 18 April 2019 due to space‐occupying lesions in the esophagogastric junction. Contrast‐enhanced CT of his abdomen demonstrated that the gastric wall was thickened by about 28 mm, and several small lymph nodes (20 mm) scattered around the stomach were detected (Fig. 5). The baseline tissue sample was identified as MLH1 (+), MSH2 (+), MSH6 (+), and PMS2 (−) by immunohistochemistry and microsatellite instability (MSI‐H) and tumor mutation burden high by NGS. After 2 cycles of SOX NACT, the gastric wall was thickened by about 33 mm, with lymph nodes enlarged 28 × 23 mm. Due to PD, the treatment scheme was modified to immunotherapy and chimeric antigen receptor T‐cell (CAR‐T) therapy. After 4 cycles of programmed cell death 1 (PD‐1) inhibitors and 3 cycles of CAR‐T administration, the gastric wall was thickened by about 20 mm with lymph nodes enlarged 21 × 20 mm. The reexamination results showed that the patients benefited from the immunotherapy. After 8 and 12 cycles of PD‐1 administration on 5 Dec 2019 and 15 Apr 2020, the disease remains stable. As of Dec 2021, the patient became resistant to immunotherapy and died.

## 4 Discussion

In this study, we examined the role of ctDNA as a predictive biomarker for response and outcome in the neoadjuvant setting. The cohort included stage II–III GC patients who were treated with standard NACT followed by D2 surgery.

ctDNA studies in resectable GC have recently been reported. Wo et al. [20] found that detectable ctDNA after chemoradiation and postoperatively was associated with disease recurrence. However, their ctDNA analysis was based on digital PCR, which limits the number of mutations that can be tested. Yang et al. showed that a ctDNA assay could detect postoperative MRD and preceded radiographic recurrence by a median of 6 months. GC patients with positive ctDNA in the postsurgical period had a worse disease‐free survival (DFS) and OS [21]. However, the patients enrolled in their study did not receive NACT but underwent gastrectomy with curative intent, followed by adjuvant chemotherapy (SOX). In the present study, the decline of the max‐VAF and the number of variants of ctDNA after NACT suggest a good response, and the persistent negative of ctDNA after NACT or surgery prompts a longer OS time. The clearance of ctDNA could not predict the efficacy of NACT alone, possibly because the patients enrolled in this study received two cycles of downstaging and downsizing intended NACT, which could only kill a portion of tumor cells. None of the patients achieved a complete response. Those residual tumor cells could still release ctDNA into the peripheral blood. This could also partially explain the decline of the max‐VAF and the number of GAs after NACT.

According to the available evidence, MSI‐high status was a favorable prognostic and potential negative predictive factor for neoadjuvant/adjuvant chemotherapy in resectable GC. Patients with MSI‐low/microsatellite stability GC could benefit from chemotherapy plus surgery: the 5‐year DFS compared with surgery only was 57% vs 41%, and the 5‐year OS was 62% vs 53% [22]. Meanwhile, MSI was associated with longer DFS (hazard ratio [HR], 1.88) and OS (HR, 1.78) [22]. MSI GCs hypermutated phenotype triggers immunosurveillance, making this molecular subgroup a promising candidate for immune checkpoint inhibitor treatment [23]. The different response of patient *#*40 to NACT and immunotherapy was consistent with previous studies and under the current knowledge in the field.

Gastric cancer was a molecularly and phenotypically highly heterogeneous disease [24, 25]. In this study, there were significant differences in GAs landscape between tissue and plasma, and before and after NACT. After excluding the experimental and sequencing errors, GAs that were present in plasma but not in tissue could be attributed to intra‐tumoral, inter‐metastatic, and intra‐metastatic heterogeneity. Meanwhile, the effects of selective therapeutic pressure of NACT may aggravate the heterogeneity in GC [26]. ctDNA used in conjunction with tissue may be an approach to overcome intra‐patient heterogeneity.

In this study, half of the patients with post‐NACT PD harbored *STK11*, *SETBP1*, or *MECOM* mutations. These GAs may participate in the development of NACT resistance and worse outcomes. *STK11* is a tumor suppressor gene that plays a role in tumorigenesis and cancer progression. Its expression was decreased in GC [27], and this positively correlated with epithelial‐mesenchymal transition and a shorter DFS [28]. Previous studies have shown that *STK11* was an upstream kinase involved in AMPK activation [29] and associated with the inhibition of tumor cell epithelial‐mesenchymal transition through the p38‐mitogen‐activated protein kinase (MAPK) signaling pathway [30]. STK11 could stimulate BRCA1 expression and stabilize BRCA1 mRNA. Similar to cells lacking BRCA1, cells lacking STK11 display increased genomic instability, accumulate DNA double‐strand breaks, display defective homology‐directed DNA repair (HDR) and exhibit an increased mutation rate [31]. Its alterations were correlated with a poor prognosis regardless of therapy across many cancer types [32]. *SETBP1* somatic mutations were well studied in many clonal myeloid disorders and associated with poor outcomes in myeloid leukemia [33, 34]. Initial studies reported that *MECOM* was a direct target of SETBP1 transcriptional activity and suggested a positive feedback loop occurring between MECOM and SETBP1 [35, 36]. SETBP1 could also upregulate MAPK signaling and downregulate differentiation pathways by enhancing the NRAS gene expression signature [33]. In this study, pathway analysis of the GAs that emerged post‐NACT shows enrichment of the MAPK pathway. These results suggest that the MAPK signaling pathway may play a role in chemotherapy resistance, although the exact explanation of this phenomenon remains unclear.

To our knowledge, our work represents the most comprehensive study on ctDNA detection in the NACT setting of patients with GC. And ctDNA examination was first associated with response to NACT and survival in GC. However, there were some limitations to this study. First, this study was subjected to a limited number of patients and potential sample selection bias. Meanwhile, affected by COVID‐19, the proportion of patients completing treatment and the follow‐up rate was low. Second, a prefabricated 425 genes panel was used. This may omit some gene variants in FFPE or ctDNA and lead to false negatives and limit the generalization of the findings. Third, due to the limitation of plasma volume, the GAs detected by NGS were not validated by other detection methods. Therefore, future efforts should be directed toward prospective trials with larger sample sizes, improved techniques, and comprehensive evaluation.

## 5 Conclusions

Clinical ctDNA testing holds promise for GC patients in monitoring NACT treatment response and predicting OS. The decline of the max‐VAF and the number of variants of ctDNA after NACT suggested a good response, and the persistent positive of ctDNA after NACT or surgery prompts a shorter OS time. ctDNA used with tissue may be an approach to overcome intra‐patient heterogeneity and could guide stratifying GC patients for NACT.

## Conflict of interest

The authors declare no conflict of interest.

## Author contributions

MZ involved in conceptualization, methodology, formal analysis, and writing—original draft; HY involved in conceptualization, investigation, and data curation; TF and MM involved in investigation, data curation, and validation; YF involved in software and Bioinformatics analysis; CQ involved in validation and investigation; ZL involved in investigation and resources; XX involved in validation and writing—review and editing; WL involved in resources and data curation; MY and SL involved in investigation and data curation; ZB involved in vonceptualization, writing—review and editing, and supervision; SJ involved in conceptualization, project administration, writing—review and editing, and funding acquisition.

## Supporting information


**Fig. S1.** The difference in genomic alteration frequency among patients with different response to NACT.

**Fig. S2.** Heterogeneity of genomic alteration in paired tissues and ctDNA before and after neoadjuvant chemotherapy.

**Fig. S3.** Kaplan–Meier estimates overall survival according to the genomic alterations in tissue.

**Fig. S4.** Kaplan–Meier estimates overall survival according to the genomic alterations in ctDNA.

**Table S1.** The 425 targeted‐panel gene list.
**Table S2.** Clinical characteristics of patients with gastric cancer.
**Table S3.** The concentration of baseline plasma cfDNA.
**Table S4.** The detectable rate of baseline plasma ctDNA.
**Table S5.** The frequency of mutated genes on gastroscope biopsy tissue between patients with detectable or undetectable ctDNA before neoadjuvant chemotherapy.
**Table S6.** The dynamic change of ctDNA in patients with various response to NACT.

## Data Availability

The datasets used and/or analyzed during the current study are available from the corresponding author upon reasonable request.
