# Supplementary material for: Liquid biopsy: circulating tumor DNA monitors neoadjuvant chemotherapy response and prognosis in stage II/III gastric cancer
Source: Mol Oncol. 2023 Jul 4;17(9):1930–42. doi: 10.1002/1878-0261.13481 (PMC10483607; doi:10.1002/1878-0261.13481)
Supplement: Supplementary file 5 — Table S1. The 425 targeted‐panel gene list. Table S2. Clinical characteristics of patients with gastric cancer. Table S3. The concentration of baseline plasma cfDNA. Table S4. The detectable rate of baseline plasma ctDNA. Table S5. The frequency of mutated genes on gastroscope biopsy tissue between patients with detectable or undetectable ctDNA before neoadjuvant chemotherapy. Table S6. The dynamic change of ctDNA in patients with various response to NACT. [file MOL2-17-1930-s005.docx]

**Supplemental Information**

*Article title:* Liquid biopsy: circulating tumor DNA monitors neoadjuvant chemotherapy response and prognosis in stage II/III gastric cancer.

*Author names:* Meng Zhang; Heli Yang; Tao Fu; Meizhu Meng; Yi Feng; Changda Qu; Zhongwu Li; Xiaofang Xing; Wenmei Li; Meiying Ye; Sisi Li; Zhaode Bu; Shuqin Jia

*Affiliation and e-mail address of the corresponding author:* Professor Shuqin Jia, email: shuqin_jia@hsc.pku.edu.cn, department of molecular diagnostics, and Professor Zhaode Bu, email: buzhaode@cjcrcn.org, Gastrointestinal Cancer Center, Peking University Cancer Hospital & Institute (Ministry of Education / Beijing), No.52 Fucheng Road, Haidian District, Beijing, 100142, China, Work telephone number: +8610 88121122

**Supplementary table 1 The 425 targeted-panel gene list**

Single nucleotide variations (SNVs) and insertions/deletions (InDels) were identified in 425 genes

| *ABCB1* | *ABCC2* | *ADGRB3* | *ADH1B* | *AIP* | *AKT1* | *AKT2* |
| --- | --- | --- | --- | --- | --- | --- |
| *AKT3* | *ALDH2* | *ALK* | *AMER1* | *APC* | *AR* | *ARAF* |
| *ARID1A* | *ARID1B* | *ARID2* | *ARID5B* | *ASCL4* | *ASXL1* | *ATF1* |
| *ATIC* | *ATM* | *ATR* | *ATRX* | *AURKA* | *AURKB* | *AXIN2* |
| *AXL* | *B2M* | *BAD* | *BAK1* | *BAP1* | *BARD1* | *BAX* |
| *BCL2* | *BCL2L11* | *BCR* | *BIRC3* | *BLM* | *BMPR1A* | *BRAF* |
| *BRCA1* | *BRCA2* | *BRD4* | *BRIP1* | *BTG2* | *BTK* | *BUB1B* |
| *c11orf30* | *CASP8* | *CBL* | *CBLB* | *CCND1* | *CCNE1* | *CD274* |
| *CD74* | *CDA* | *CDC73* | *CDH1* | *CDK10* | *CDK12* | *CDK4* |
| *CDK6* | *CDK8* | *CDKN1A* | *CDKN1B* | *CDKN1C* | *CDKN2A* | *CDKN2B* |
| *CDKN2C* | *CEBPA* | *CEP57* | *CHD4* | *CHD8* | *CHEK1* | *CHEK2* |
| *CREBBP* | *CRKL* | *CSF1R* | *CTCF* | *CTLA4* | *CTNNB1* | *CUL3* |
| *CUX1* | *CXCR4* | *CYLD* | *CYP19A1* | *CYP2A13* | *CYP2A6* | *CYP2A7* |
| *CYP2B6*6* | *CYP2C19*2* | *CYP2C9*3* | *CYP2D6* | *CYP3A4*4* | *CYP3A5* | *CYSLTR2* |
| *DAXX* | *DDR2* | *DENND1A* | *DHFR* | *DICER1* | *DLL3* | *DNMT3A* |
| *DOT1L* | *DPYD* | *DTL(CDT2)* | *DUSP2* | *EGFR* | *EIF1AX* | *EP300* |
| *EPAS1* | *EPCAM* | *EPHA2* | *EPHA3* | *EPHA5* | *ERBB2* | *ERBIN* |
| *ERBB3* | *ERBB4* | *ERCC1* | *ERCC2* | *ERCC3* | *ERCC4* | *ERCC5* |
| *ESR1* | *ETV1* | *ETV4* | *ETV5* | *ETV6* | *EWSR1* | *EXT1* |
| *EXT2* | *EZH2* | *EZR* | *FANCA* | *FANCC* | *FANCD2* | *FANCE* |
| *FANCF* | *FANCG* | *FANCI* | *FANCL* | *FANCM* | *FAT1* | *FBXW7* |
| *FGF19* | *FGFR1* | *FGFR2* | *FGFR3* | *FGFR4* | *FH* | *FLCN* |
| *FLT1* | *FLT3* | *FLT4* | *FOXA1* | *FOXL2* | *FOXP1* | *FRG1* |
| *GATA1* | *GATA2* | *GATA3* | *GATA4* | *GATA6* | *GNA11* | *GNAQ* |
| *GNAS* | *GRIN2A* | *GRM3* | *GRM8* | *GSTM1* | *GSTM4* | *GSTP1* |
| *GSTT1* | *HDAC2* | *HDAC9* | *HGF* | *HLA-A* | *HNF1A* | *HNF1B* |
| *HRAS* | *IDH1* | *IDH2* | *IFNA6* | *IFNB1* | *IFNE* | *IFNG* |
| *IFNGR1* | *IFNGR2* | *IGF1R* | *IGF2* | *IKBKE* | *IKZF1* | *IL7R* |
| *INPP4B* | *IRF2* | *JAK1* | *JAK2* | *JAK3* | *JARID2* | *JUN* |
| *KDM5A* | *KDR* | *KEAP1* | *KIF1B* | *KIT* | *KITLG* | *KLLN* |
| *KMT2A* | *KMT2B* | *KMT2C* | *KMT2D* | *KRAS* | *LHCGR* | *LMO1* |
| *LRP1B* | *LYN* | *LZTR1* | *MAP2K1* | *MAP2K2* | *MAP2K4* | *MAP3K1* |
| *MAP3K4* | *MAX* | *MCL1* | *MDM2* | *MDM4* | *MECOM* | *MED12* |
| *MEF2B* | *MEN1* | *MET* | *MGMT* | *MITF* | *MLH1* | *MLH3* |
| *MLLT1* | *MLLT3* | *MLLT4* | *MPL* | *MRE11* | *MSH2* | *MSH6* |
| *MTHFR* | *MTOR* | *MUTYH* | *MYC* | *MYCL* | *MYCN* | *MYD88* |
| *MYH9* | *NAT1* | *NBN* | *NCOR1* | *NF1* | *NF2* | *NFE2L2* |
| *NFKBIA* | *NKX2-1* | *NOTCH1* | *NOTCH2* | *NOTCH3* | *NPM1* | *NQO1* |
| *NRAS* | *NRG1* | *NSD1* | *NTRK1* | *NTRK2* | *NTRK3* | *NUTM1* |
| *PAK3* | *PALB2* | *PALLD* | *PARK2* | *PARP1* | *PARP2* | *PAX5* |
| *PBRM1* | *PDCD1* | *PDCD1LG2* | *PDE11A* | *PDGFRA* | *PDGFRB* | *PDK1* |
| *PGR* | *PHOX2B* | *PIK3C3* | *PIK3CA* | *PIK3CD* | *PIK3R1* | *PIK3R2* |
| *PKHD1* | *PLAG1* | *PLCB4* | *PLK1* | *PMS1* | *PMS2* | *POLD1* |
| *POLD3* | *POLE* | *POLH* | *POT1* | *PPARD* | *PPP2R1A* | *PRDM1* |
| *PREX2* | *PRF1* | *PRKACA* | *PRKAR1A* | *PRKCI* | *PRKDC* | *PRSS1* |
| *PRSS3* | *PTCH1* | *PTEN* | *PTK2* | *PTPN11* | *PTPN13* | *QKI* |
| *RAC1* | *RAC3* | *RAD50* | *RAD51* | *RAD51B* | *RAD51C* | *RAD51D* |
| *RAD54L* | *RAF1* | *RARA* | *RARG* | *RASGEF1A* | *RB1* | *RECQL4* |
| *RELN* | *RET* | *RHOA* | *RICTOR* | *RNF43* | *ROS1* | *RPTOR* |
| *RRM1* | *RUNX1* | *RUNX1T1* | *SBDS* | *SDC4* | *SDHA* | *SDHB* |
| *SDHC* | *SDHD* | *SEPTIN9* | *SETBP1* | *SETD2* | *SF3B1* | *SGK1* |
| *SKP2* | *SLC34A2* | *SLC3A2* | *SMAD2* | *SMAD3* | *SMAD4* | *SMAD7* |
| *SMARCA4* | *SMARCB1* | *SMO* | *SOCS1* | *SOS1* | *SOX2* | *SPOP* |
| *SPRED1* | *SPRY4* | *SRC* | *SRSF2* | *SRY* | *STAG2* | *STAT3* |
| *STK11* | *STMN1* | *SUFU* | *TACC3* | *TAP1* | *TAP2* | *TEK* |
| *TEKT4* | *TERC* | *TERT* | *TET2* | *TGFBR2* | *THADA* | *TMEM127* |
| *TMPRSS2* | *TNFAIP3* | *TNFRSF11A* | *TNFRSF14* | *TNFRSF19* | *TNFSF11* | *TOP1* |
| *TOP2A* | *TP53* | *TP63* | *TPMT* | *TSC1* | *TSC2* | *TSHR* |
| *TTF1* | *TUBB3* | *TYMS* | *U2AF1* | *UGT1A1* | *VAMP2* | *VEGFA* |
| *VHL* | *WAS* | *WISP3* | *WRN* | *WT1* | *XPA* | *XPC* |
| *XRCC1* | *XRCC2* | *YAP1* | *ZNF217* | *ZNF703* |  |  |

Structural variations (SV) were identified in 57 genes

| *AKT2* | *ALK* | *AXL* | *BCL2* | *BCR* | *BIRC3* | *BRAF* |
| --- | --- | --- | --- | --- | --- | --- |
| *BRCA1* | *BRCA2* | *BRD4* | *CCND1* | *CD274* | *CD74* | *CDK12* |
| *CDK4* | *CDKN2A* | *EGFR* | *ERBB2* | *ERBB3* | *ETV1* | *ETV4* |
| *ETV6* | *EWSR1* | *EZR* | *FGFR1* | *FGFR2* | *FGFR3* | *JAK2* |
| *KIT* | *KMT2A* | *MET* | *MYC* | *NOTCH1* | *NOTCH2* | *NPM1* |
| *NRG1* | *NTRK1* | *NTRK2* | *NTRK3* | *NUTM1* | *PDGFRA* | *PDGFRB* |
| *PRKACA* | *RAD50* | *RAF1* | *RARA* | *RELN* | *RET* | *ROS1* |
| *RUNX1* | *RUNX1T1* | *SDC4* | *SLC34A2* | *TACC3* | *TMPRSS2* | *TP53* |
| *YAP1* |  |  |  |  |  |  |

Copy number variations (CNV) were identified in 103 genes

| *ABCB1* | *AKT1* | *AKT2* | *AKT3* | *ALK* | *APC* | *AR* |
| --- | --- | --- | --- | --- | --- | --- |
| *AURKB* | *AXL* | *BCL2* | *BMPR1A* | *BRAF* | *CCND1* | *CCNE1* |
| *CD274* | *CDH1* | *CDK12* | *CDK4* | *CDK6* | *CDKN2A* | *CDKN2B* |
| *CRKL* | *CSF1R* | *DDR2* | *DLL3* | *EGFR* | *EPCAM* | *ERBB2* |
| *ERBB3* | *ESR1* | *FGF19* | *FGFR1* | *FGFR2* | *FGFR3* | *FGFR4* |
| *FLT1* | *FLT3* | *FLT4* | *GNAS* | *HGF* | *HRAS* | *IGF1R* |
| *IGF2* | *IL7R* | *JAK1* | *KIT* | *KRAS* | *MAP2K1* | *MAP2K2* |
| *MCL1* | *MDM2* | *MDM4* | *MET* | *MGMT* | *MLH1* | *MSH2* |
| *MSH6* | *MTOR* | *MUTYH* | *MYC* | *MYCN* | *NF1* | *NKX2-1* |
| *NOTCH2* | *NRAS* | *NTRK1* | *PDCD1LG2* | *PDGFRA* | *PDGFRB* | *PIK3CA* |
| *PMS2* | *POLD1* | *POLE* | *PTEN* | *PTK2* | *RB1* | *RET* |
| *RICTOR* | *RRM1* | *SDHA* | *SDHB* | *SDHC* | *SDHD* | *SMAD4* |
| *SMARCB1* | *SMO* | *SOX2* | *SRC* | *STK11* | *STMN1* | *TERC* |
| *TERT* | *TOP1* | *TOP2A* | *TP53* | *TSC1* | *TSC2* | *TTF1* |
| *TUBB3* | *VEGFA* | *VHL* | *ZNF217* | *ZNF703* |  |  |

**Supplementary table 2 Clinical characteristics of patients with gastric cancer**

| Characteristics | Total evaluable cohort, *N* (%) |
| --- | --- |
| Age (years) |  |
| Median (IQR) | 60 (52-65) |
| Sex |  |
| Female | 23 (29.1) |
| Male | 56 (70.9) |
| Smoking |  |
| No | 41 (51.9) |
| Yes | 28 (35.4) |
| Unknown | 10 (12.7) |
| Drinking |  |
| No | 49 (62.0) |
| Yes | 20 (25.3) |
| Unknown | 10 (12.7) |
| Stage |  |
| II | 22 (27.8) |
| III | 57 (72.2) |
| Histological grade |  |
| Moderately | 20 (25.3) |
| Moderately & poorly | 15 (19.0) |
| Poorly | 43 (54.4) |
| Unknown | 1 (1.3) |
| Lauren type |  |
| Intestinal | 35 (44.3) |
| Mixed | 15 (19.0) |
| Diffuse | 27 (34.1) |
| Unknown | 2 (2.6) |
| Response to NACT |  |
| PD | 5 (6.3) |
| SD | 45 (57.0) |
| PR | 25 (31.6) |
| Unknown | 4 (5.0) |

**Supplementary table 3 The concentration of baseline plasma cfDNA**

| Characteristics | P0 concentration  (ng/mL) | *P* value |
| --- | --- | --- |
| Age (years) |  |  |
| <60 | 16.19±5.80 | 0.740 |
| ≥60 | 14.12±5.79 |  |
| Sex |  |  |
| Female | 14.53±5.90 | 0.850 |
| Male | 16.63±5.57 |  |
| Smoking |  |  |
| No | 15.66±6.40 | 0.435 |
| Yes | 13.96±5.52 |  |
| Drinking |  |  |
| No | 15.12±6.44 | 0.259 |
| Yes | 14.58±5.22 |  |
| Stage |  |  |
| II | 14.40±7.34 | **0.005** |
| III | 15.43±5.21 |  |
| Histological grade |  |  |
| Moderately | 15.96±4.83 | 0.798 |
| Moderately & poorly | 14.78±7.28 |  |
| Poorly | 15.00±5.5.86 |  |
| Lauren type |  |  |
| Intestinal | 15.18±4.80 | 0.927 |
| Mixed | 15.07±7.13 |  |
| Diffuse | 15.68±6.35 |  |
| Response to NACT^1^ |  |  |
| PR | 16.61±6.54 | **0.009** |
| SD | 13.57±5.25 |  |
| PD | 20.68±3.75 |  |
| Detectable |  |  |
| Yes | 15.91±6.17 | 0.496 |
| No | 14.01±5.23 |  |

^1^ Inter-group comparisons were done using Scheffe post hoc multiple comparisons test. Patients in PD group have a significantly higher mean concentration of ctDNA than SD group (P=0.033), but have no difference with PR group (P=0.344)

**Supplementary table 4 The detectable rate of baseline plasma ctDNA**

| Characteristics | P0 undetectable  *N* (%) | P0 detectable  *N* (%) | *P* value |
| --- | --- | --- | --- |
| Age (years) |  |  |  |
| <60 | 18 (46.2%) | 21 (53.8%) | 0.313 |
| ≥60 | 14 (35.0%) | 26 (65.0%) |  |
| Sex |  |  |  |
| Female | 9 (39.1%) | 14 (60.9%) | 0.873 |
| Male | 23 (41.1%) | 33 (58.9%) |  |
| Smoking |  |  |  |
| No | 16 (39.0%) | 25 (61.0%) | 0.366 |
| Yes | 14 (50.0%) | 14 (50.0%) |  |
| Unknown | 2 (20.0%) | 8 (80.0%) |  |
| Drinking |  |  |  |
| No | 21 (42.9%) | 28 (57.1%) | 0.871 |
| Yes | 9 (45.0%) | 11 (55.0%) |  |
| Unknown | 0 (0.0%) | 8 (100.0%) |  |
| Stage |  |  |  |
| II | 12 (54.5%) | 10 (45.5%) | 0.114 |
| III | 20 (35.1%) | 37 (64.9%) |  |
| Histological grade |  |  |  |
| Moderately | 7 (35.0%) | 13 (65.0%) | 0.783 |
| Moderately & poorly | 7 (46.7%) | 8 (53.3%) |  |
| Poorly | 17 (39.5%) | 26 (60.5%) |  |
| Unknown | 1 (100.0%) | 0 (0.0%) |  |
| Lauren type |  |  |  |
| Intestinal | 13 (37.1%) | 22 (62.9%) | 0.246 |
| Mixed | 4 (26.7%) | 11 (73.3%) |  |
| Diffuse | 14 (51.9%) | 13 (48.1%) |  |
| Unknown | 1 (50.0%) | 1 (50.0%) |  |
| Response to NACT |  |  |  |
| PD | 0 (0.0%) | 5 (100.0%) | **0.011** |
| SD | 25 (55.6%) | 20 (44.4%) |  |
| PR | 7 (28.0%) | 18 (72.0%) |  |
| Unknown | 0 (0.0%) | 4 (100.0%) |  |

**Supplementary table 5. The frequency of mutated genes on gastroscope biopsy tissue between patients with** **detectable or undetectable ctDNA before neoadjuvant chemotherapy.**

|  | ctDNA detectable | | ctDNA undetectable | |  |
| --- | --- | --- | --- | --- | --- |
| Gene | Mutated | Wildtype | Mutated | Wildtype | *P* value^*^ |
| ADGRB3 | 0 | 27 | 2 | 15 | 0.144 |
| AR | 3 | 24 | 1 | 16 | 1.000 |
| ARID1A | 5 | 22 | 6 | 11 | 0.289 |
| ARID2 | 1 | 26 | 2 | 15 | 0.549 |
| ATR | 0 | 27 | 2 | 15 | 0.144 |
| BRCA1 | 2 | 25 | 0 | 17 | 0.515 |
| BRCA2 | 2 | 25 | 1 | 16 | 1.000 |
| CBLB | 0 | 27 | 2 | 15 | 0.144 |
| CCNE1 | 4 | 23 | 1 | 16 | 0.634 |
| CDH1 | 5 | 22 | 4 | 13 | 0.716 |
| CDKN2A | 3 | 24 | 0 | 17 | 0.272 |
| CHD4 | 3 | 24 | 0 | 17 | 0.272 |
| CREBBP | 4 | 23 | 1 | 16 | 0.634 |
| DOT1L | 3 | 24 | 1 | 16 | 1.000 |
| DPYD | 4 | 23 | 0 | 17 | 0.147 |
| EGFR | 2 | 25 | 1 | 16 | 1.000 |
| EP300 | 1 | 26 | 2 | 15 | 0.549 |
| ERBB2 | 3 | 24 | 0 | 17 | 0.272 |
| ERBB3 | 2 | 25 | 0 | 17 | 0.515 |
| ERBB4 | 2 | 25 | 0 | 17 | 0.515 |
| EZH2 | 2 | 25 | 0 | 17 | 0.515 |
| FANCC | 2 | 25 | 0 | 17 | 0.515 |
| FANCE | 2 | 25 | 0 | 17 | 0.515 |
| FANCI | 2 | 25 | 0 | 17 | 0.515 |
| FAT1 | 2 | 25 | 0 | 17 | 0.515 |
| FBXW7 | 2 | 25 | 1 | 16 | 1.000 |
| FGFR2 | 3 | 24 | 1 | 16 | 1.000 |
| FLT3 | 2 | 25 | 0 | 17 | 0.515 |
| GNAS | 3 | 24 | 3 | 14 | 0.662 |
| GRIN2A | 2 | 25 | 0 | 17 | 0.515 |
| GRM3 | 2 | 25 | 2 | 15 | 0.634 |
| HGF | 4 | 23 | 0 | 17 | 0.147 |
| JAK3 | 1 | 26 | 2 | 15 | 0.549 |
| KMT2A | 0 | 27 | 2 | 15 | 0.144 |
| KMT2B | 3 | 24 | 1 | 16 | 1.000 |
| KRAS | 4 | 23 | 1 | 16 | 0.634 |
| LRP1B | 6 | 21 | 4 | 13 | 1.000 |
| MAP2K1 | 1 | 26 | 2 | 15 | 0.549 |
| MCL1 | 2 | 25 | 0 | 17 | 0.515 |
| MECOM | 3 | 24 | 0 | 17 | 0.272 |
| MLH3 | 2 | 25 | 0 | 17 | 0.515 |
| MYC | 4 | 23 | 1 | 16 | 0.634 |
| NOTCH1 | 4 | 23 | 0 | 17 | 0.147 |
| NOTCH2 | 3 | 24 | 0 | 17 | 0.272 |
| NRG1 | 3 | 24 | 1 | 16 | 1.000 |
| NSD1 | 3 | 24 | 0 | 17 | 0.272 |
| PDCD1LG2 | 2 | 25 | 0 | 17 | 0.515 |
| PIK3CA | 4 | 23 | 3 | 14 | 1.000 |
| PKHD1 | 2 | 25 | 0 | 17 | 0.515 |
| POLE | 2 | 25 | 1 | 16 | 1.000 |
| PREX2 | 2 | 25 | 3 | 14 | 0.359 |
| PTEN | 2 | 25 | 0 | 17 | 0.515 |
| RECQL4 | 2 | 25 | 1 | 16 | 1.000 |
| RET | 2 | 25 | 1 | 16 | 1.000 |
| RNF43 | 0 | 27 | 2 | 15 | 0.144 |
| ROS1 | 2 | 25 | 0 | 17 | 0.515 |
| RPTOR | 2 | 25 | 0 | 17 | 0.515 |
| SETBP1 | 3 | 24 | 0 | 17 | 0.272 |
| SETD2 | 3 | 24 | 2 | 15 | 1.000 |
| SMAD4 | 5 | 22 | 0 | 17 | 0.139 |
| SMARCA4 | 3 | 24 | 1 | 16 | 1.000 |
| STK11 | 2 | 25 | 1 | 16 | 1.000 |
| TERC | 2 | 25 | 0 | 17 | 0.515 |
| TGFBR2 | 2 | 25 | 0 | 17 | 0.515 |
| TP53 | 19 | 8 | 8 | 9 | 0.203 |
| ZNF217 | 2 | 25 | 0 | 17 | 0.515 |

^*^The frequency of all tested genes with alterations of more than one in patients with either detectable or undetectable ctDNA was examined by Fisher’s exact test.

**Supplementary table 6. The** **dynamic change of ctDNA in patients with various response to NACT**

|  | P1 negative | |  | P1 negative | | P value |
| --- | --- | --- | --- | --- | --- | --- |
|  | P0 negative | P0 positive |  | P0 negative | P0 positive |  |
| PR | 5 (23.8%) | 6 (28.6%) |  | 1 (4.8%) | 9 (42.9%) | 0.231 |
| SD | 15 (36.6%) | 6 (14.6%) |  | 7 (17.1%) | 13 (31.7%) |  |
| PD | 0 (0.0%) | 0 (0.0%) |  | 0 (0.0%) | 3 (100.0%) |  |

**Supplementary figure legend**

**Supplementary Figure 1 The difference in genomics alteration frequency among patients with different responses to NACT.**The depth of red color indicated the proportion of patients carrying a certain altered gene in different groups of response to NACT.

**Supplementary Figure 2 Heterogeneity of genomic alteration in paired tissues and ctDNA before and after neoadjuvant chemotherapy.** Venn diagram identifying the mutated genes (A) and GAs (B) among matched tissue and plasma before and after NACT. (C) GO pathway analyses of GAs that vanished (red) and appeared (blue) after NATC.

**Supplementary Figure 3 Kaplan-Meier estimates overall survival according to the genomic alterations in tissue.** The Gehan-Breslow-Wilcoxon test was used for comparison of the survival curve

**Supplementary Figure 4 Kaplan-Meier estimates overall survival according to the genomic alterations in ctDNA.** The Gehan-Breslow-Wilcoxon test was used for comparison of the survival curve.
